# Genetic Analyses of the Internal Transcribed Spacer Sequences Suggest Introgression and Duplication in the Medicinal Mushroom *Agaricus subrufescens*

**DOI:** 10.1371/journal.pone.0156250

**Published:** 2016-05-26

**Authors:** Jie Chen, Magalie Moinard, Jianping Xu, Shouxian Wang, Marie Foulongne-Oriol, Ruilin Zhao, Kevin D. Hyde, Philippe Callac

**Affiliations:** 1 INRA, MycSA, Villenave d’Ornon, France; 2 Center of Excellence in Fungal Research, Mae Fah Luang University, Chiang Rai, Thailand; 3 School of Science, Mae Fah Luang University, Chiang Rai, Thailand; 4 Department of Biology, McMaster University, Hamilton, Ontario, Canada; 5 Institute of Plant and Environment Protection, Beijing Academy of Agriculture and Forestry Sciences, Beijing, China; 6 Beijing Engineering Research Center for Edible Mushroom, Beijing, China; 7 State Key Laboratory of Mycology, Institute of Microbiology, Chinese Academy of Sciences, Beijing, China; University of Sydney, AUSTRALIA

## Abstract

The internal transcribed spacer (ITS) region of the nuclear ribosomal RNA gene cluster is widely used in fungal taxonomy and phylogeographic studies. The medicinal and edible mushroom *Agaricus subrufescens* has a worldwide distribution with a high level of polymorphism in the ITS region. A previous analysis suggested notable ITS sequence heterogeneity within the wild French isolate CA487. The objective of this study was to investigate the pattern and potential mechanism of ITS sequence heterogeneity within this strain. Using PCR, cloning, and sequencing, we identified three types of ITS sequences, A, B, and C with a balanced distribution, which differed from each other at 13 polymorphic positions. The phylogenetic comparisons with samples from different continents revealed that the type C sequence was similar to those found in Oceanian and Asian specimens of *A*. *subrufescens* while types A and B sequences were close to those found in the Americas or in Europe. We further investigated the inheritance of these three ITS sequence types by analyzing their distribution among single-spore isolates from CA487. In this analysis, three co-dominant markers were used firstly to distinguish the homokaryotic offspring from the heterokaryotic offspring. The homokaryotic offspring were then analyzed for their ITS types. Our genetic analyses revealed that types A and B were two alleles segregating at one locus *ITSI*, while type C was not allelic with types A and B but was located at another unlinked locus *ITSII*. Furthermore, type C was present in only one of the two constitutive haploid nuclei (*n*) of the heterokaryotic (*n+n*) parent CA487. These data suggest that there was a relatively recent introduction of the type C sequence and a duplication of the ITS locus in this strain. Whether other genes were also transferred and duplicated and their impacts on genome structure and stability remain to be investigated.

## Introduction

The internal transcribed spacer (ITS) region of nuclear ribosomal DNA (rDNA) has been proposed as a universal DNA barcode marker for fungi [1]. It is widely used in fungal taxonomy and phylogeny due to a high success rate in PCR amplification and a high degree of interspecific variation that can distinguish between most of the closely related fungal species. It is generally believed that multicopy genes such as the nuclear ribosomal RNA gene cluster undergo frequent concerted evolutionary process, making the copies identical or highly similar to each other [2–5]. The two most commonly proposed mechanisms for concerted evolution are gene conversion [6] and unequal crossing-over [7]. However, numerous studies revealed that the multicopy genes, such as ITS, do not perfectly follow the concerted evolution, and exhibit intra-strain and intra-species variation in a wide variety of organisms [3, 5, 8]. For example, intra-strain or intra-species variation in ITS sequences have been reported in several fungal genera and species such as *Fusarium* [9], *Scutellospora* [10], *Ganoderma* [11], *Xanthophyllomyces* [12], *Laetiporus* [4] and more recently in *Ophiocordyceps sinensi*s [5].

*Agaricus subrufescens* Peck, the “almond mushroom”, is a species commercially cultivated for its nutritional and medicinal values [13–15]. This species has been recorded from different continents: the Americas, Asia, Europe, Oceania [13, 16], and recently in Africa [17].Thongklang et al. [16] showed that isolates of *A*. *subrufescens* from Asia, Europe and South America were inter-fertile and had an amphithallic life cycle (i.e. with both the heterothallic and pseudohomothallic life cycles) where both homokaryotic and heterokaryotic spores can be produced in the same sporocarp. *Agaricus subrufescens* is a morphologically and genetically highly variable species with a high level polymorphism in the ITS regions [13]. Direct PCR and sequencing of strains with a high level of intragenomic ITS polymorphism often yield chromatographs with many heterozygous sites [13]. Chromatographs with many heterozygous sites have also been reported in other species of the genus *Agaricus* [18] and other Basidiomycota such as *Hebeloma aminophilum* [19].

**Fig 1:**
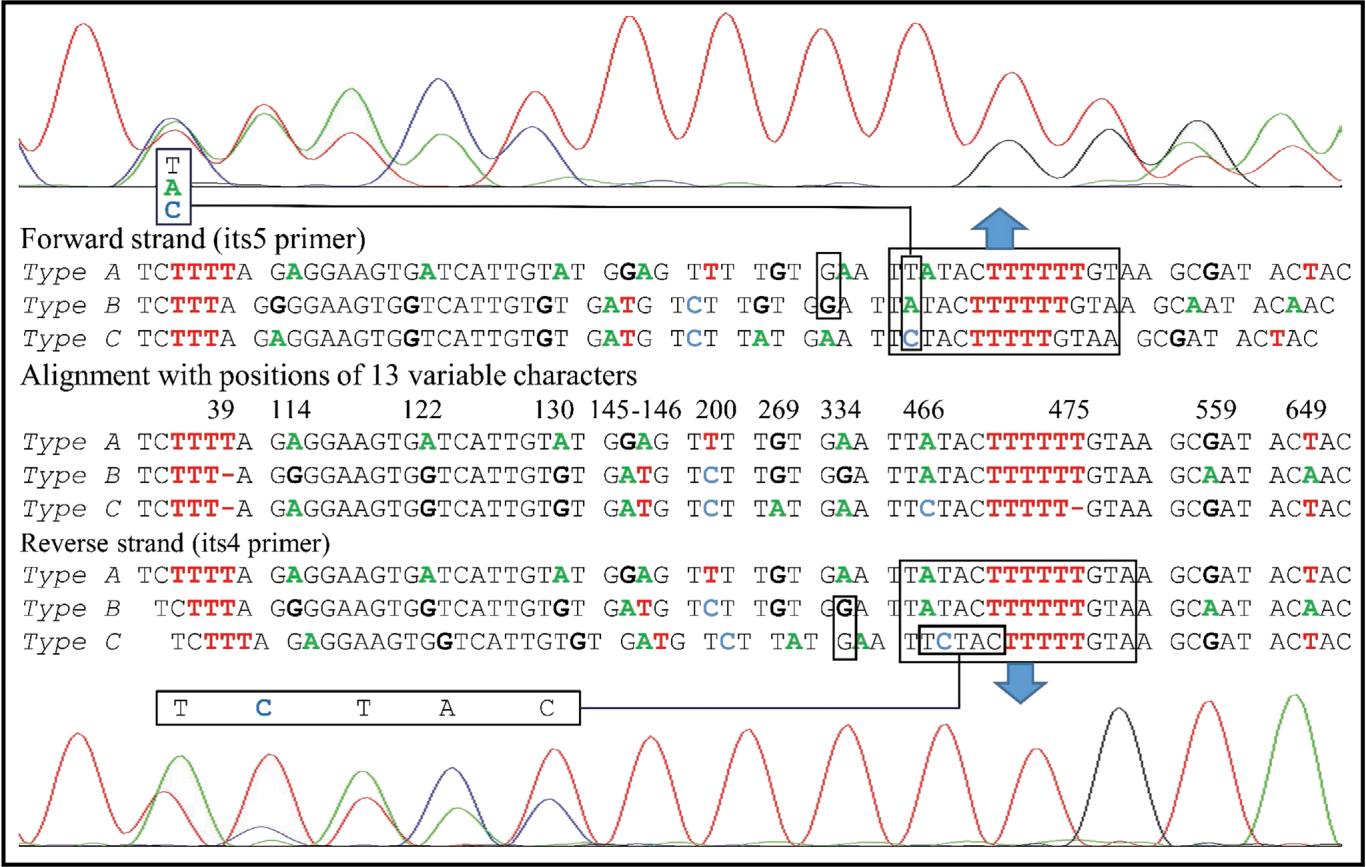
Double or triple peaks observed in the chromatogram of the ITS region of the parental isolate CA487.

The wild isolate of *A*. *subrufescens* CA487 collected at Saint-Leon, France, is one of the specimens previously studied by Thongklang et al. [16]. As in many specimens of this species the ITS sequence of CA487 was highly heterozygous with the sequences of two homokaryotic single spore isolates CA487-S100 and CA487-S42 (GenBank accession numbers KJ541799 and KJ541800 respectively) differing at ten nucleotide sites. However, after reexamining the chromatogram of the ITS region of the parental isolate CA487, we noted that differences between the two progeny could not explain several sites with double peaks and triple peaks (Fig 1).

Here we tested the hypothesis that the ITS chromatogram of isolate CA487 consists of three different types of ITS. If the hypothesis was correct, we further investigated the putative origins of the divergent ITS sequences by comparing our ITS sequences with those from other sources. In addition, we examined the inheritance patterns of the divergent ITS sequences. Our results indicate that there are indeed three equally abundant ITS sequences within this strain, with two types of ITS (A and B) segregating as two alleles at a first locus ‘*ITSI*’, while a third non-allelic type of ITS (C) was located at a second unlinked locus ‘*ITSII*’. Interestingly, the types A and B sequences were closely related to those from Europe and the Americas and the type ‘C’ was similar to ITS sequences found in Asian and Oceanian specimens of the species.

## Material and Methods

### Field collections and single spore isolates

The strain CA487 was isolated by tissue culture from a field specimen collected by J.C. Blanchard in his own garden at Saint-Leon (20 km from Bordeaux), France, on September 28^th^, 2006 (identified by J. Guinberteau). A second specimen CA516 was collected by P. Callac and J. Guinberteau at the same site on October 20^th^, 2006.

For sporocarp production in an environmentally controlled culture room, the mycelium of strain CA487 was cultivated following the method described in Llarena-Hernández et al. [20]. Single spore isolates (SSIs) were obtained from spore prints directly from cultivated sporocarps following the method described by Thongklang et al. [16]. After spore germination, 120 monospore cultures were isolated and subcultured on compost extract medium (aqueous extract of pasteurized commercial mushroom compost plus 1% glucose and 2% agar).

In addition to the sequences obtained from strain CA487 and its SSIs, sequences from 18 specimens were used for sequence comparison. Among them, 13 sequences were retrieved from GenBank and these sequences have been previously used by Kerrigan [13], Zhao et al. [21], Thongklang et al. [16] and Gui et al. [22]. Four of the 18 sequences from strains ZRL2036, ZRL2134, NT001 and CA935 were generated in previous studies [21, 23] but were not submitted until now through this work. The remaining strain CA864 was also added to our analyses for comparison; it was isolated from a wild specimen collected on distillation residue of essential oils of Thuja, at about 300 km away from where CA487 was collected. CA864 was collected by J.C. Chasles (with morphological identification by J. Guinberteau) on November 5^th^, 2010 at Saint Lézin, Chemillé-en-Anjou, France. The samples from UK and Martinique have been initially identified as *Agaricus rufotegulis* (syn. *A*. *subrufescens*) and *Agaricus fiardii*, respectively.

### DNA isolation and PCR amplification

DNA extraction was performed for 94 SSIs following the protocol described by Zhao et al. [21]. DNA concentration was adjusted to 25 ng/μl. PCR amplification was performed in 30 μl of reaction mixture containing 50 ng genomic DNA, 0.5 μM of each primer, 0.2 mM dNTPs, 2 μg BSA, 1 U Taq DNA Polymerase and 1x incubation buffer as described by Zhao et al. [21]. Sequences of the primers are indicated in Table 1.

**Table 1:** Cleaved amplified polymorphic sequence markers used to study allelic segregation of the CA487.

Sequenced DNA fragment	Locus	Genotypes	Phenotypes
and primers (5’-3’)	Endonuclease		(fragment size in bp)
	Restriction site		
PRS016:	*PRS016*:*256*	*1* or *1/1*	481+219
16F:CCGTCAAGGTCCTCAGTGAT	*SaI*I	*2* or *2/2*	271+219+210
16R:TTTAGTGCTCATGGCAGCAG	gtgaGTCGA**S**ttyt	*1/2*	481+271+219+210
PRS049:	*PRS049*:*167*	*1* or *1/1*	479
49F: CCGAACGGTTCCATGACTAT	*Kpn*I	*2* or *2/2*	292+187
49R: TGTGGTCTCGCTTCATCTTG	aaatGGTA**Y**Cttct	*1/2*	479+292+187
PRS088:	*PRS088*:*432*	*1* or *1/1*	757
88F:CTCGCAATTAGCTTCCAAGG	*Ava*II	*2* or *2/2*	449+308
88R:CGGTTGTCCAAGATCAAGGT	ttctGGT**Y**Cgtcc	*1/2*	757+449+308
ITS1+5.8S+ITS2	*ITSI-ITSII:334-466*^*a*^	*ItsI-a ItsII-n*^b^	396+377
ITS5:GGAAGTAAAAGTCGTAACAAGG	*Mbo*II	*ItsI-b ItsII-n*	772
ITS4: TCCTCCGCTTATTGATATGC	cgatGAAG**R**acgc 334	(*ItsII-*c)^c^	(395+264+112^)^^c^
	actcTCTT**M**tact 466	See *ITSI*+*ITSII* in Table 2	
	*ITSI-ITSII*:*145–146*^*a*^	*ItsI-a ItsII-n*	773
	*Fok*I	*ItsI-b ItsII-n*	563+209
	tgctGG**RW**Gtgag	(*ItsII-c*)^c^	(562+209)^c^
		See *ITSI*+*ITSII* in Table 2	

^a^ Markers *ITSI-ITSII*:*334–466* and *ITSI-ITSII*:*145–146*: all possible genotypes loci *ITSI* and *ITSII* and corresponding phenotypes are detailed in Table 2

^b^ Null allele

^c^ Genotypes and phenotypes never present or observed alone

### Cloning and sequencing

PCR products of parental isolate CA487 were purified either directly or after agarose gel electrophoresis and band excision using the QIAquick PCR purification kit (Qiagen, Hilden, Germany) or the Gel Extraction kit (Qiagen, Hilden, Germany). The purified PCR products were then ligated into a pMD19-T vector (Takara, Tokyo, Japan) at 16°C overnight. Recombined plasmids were transformed and replicated into competent cells of *Escherichia coli* strain Top10 (TianGen Biotech., Co., Ltd., Beijing, China). Plasmids containing the cloned PCR products were extracted using QIAprep Miniprep Kit (Qiagen, Hilden, Germany), then was used as template for sequencing as described in Zhao et al. [21].

### Phylogenetic analysis

The sequences produced from this study plus those retrieved from GenBank were aligned using BioEdit v. 7.0.4 [24]. The maximum parsimony (MP) analysis was performed with PAUP* 4.0b10 [25] by heuristic searches with unordered characters, random addition of sequences, gaps included as coded characters, and used tree bisection-reconnection (TBR) branch swapping for optimization. The MP analysis included 1000 bootstrap replicates.

### Map position of markers

Co-dominant markers were developed firstly to distinguish between the homokaryotic and heterokaryotic single spore isolates, and secondly to distinguish the different types of ITS present in the single spore isolates. Thongklang et al. [16] showed that in strains of *A*. *subrufescens* such as CA487, the basidia produce not only classical homokaryotic spores but also a variable percentage of heterokaryotic spores. Because at least 99% of the basidia were tetrasporic, it was hypothesized that ability to produce both types of spores was likely due to one additional round of post-meiotic mitosis occurring in a variable proportion of basidia and generating eight haploid nuclei [16, 26]. In such basidia each basidiospore receive two nuclei would be heterokaryotic. The rate of heterokaryotic spores depends on the parent strain but is also sensitive to the environmental conditions [13] and can vary from 40% to 75% [16].

In a theoretical random tetrasporic model proposed by Rocha de Brito et al. [26], the nuclei are paired at random and the expected loss rate of parental heterozygosity among the heterokaryotic offspring is 33%. This applies to any locus including the centromeres; subsequently, two-third receive non-sister nuclei (i.e. each one per second meiotic division), while the remaining one–third receive sister nuclei. The heterokaryons receiving non-sister nuclei are, therefore, highly heterozygous in the centromeric region and a co-dominant marker tightly linked to a centromere would be able to reveal heterokaryosis [26]. However, such a marker is not appropriate to identify heterokaryons containing sister nuclei, which are highly homozygous in the centromeric region. It can be noted that, in different studied offspring, Thonhklang et al. [16] and Rocha de Brito et al. [26] generally observed a loss rate of parental heterozygozity of about 50% at markers unlinked to the centromere due to crossovers, i.e. higher than the 33% expected in the theoretical model. Therefore, both centromere-linked and -unlinked markers are needed to reliably identify heterokaryotic offspring.

EST sequences of *A*. *subrufescens* identified by Foulongne-Oriol et al. [27] with putative homologs in the genome of *Agaricus bisporus* were selected. Information about their physical positions on the *A*. *bisporus* genome was used to develop informative molecular markers to distinguish between the different expected types of spores among the CA487 SSIs. First, we selected an *A*. *subrufescens* sequence (ci isotig00274, GenBank: GBEJ01008187) for which *A*. *bisporus* homolog was close to *MAT* and the centromere (PRS088 marker), and its tight linkage with the centromere was recently confirmed in *A*. *subrufescens* [26] and more generally a highly conserved macro-synteny between the two species [28]. Then, two other *A*. *subrufescens* sequences, for which *A*. *bisporus* homologs were found in distal positions on chromosomes X and III were used to develop markers PRS016 and PRS049, respectively (Table 1). PRS016 and PRS049 were derived from cl isotig01118 (GenBank: GBEJ01009029) and cl isotig1243 (GenBank: GBEJ01009154) transcribed RNA sequences, respectively.

### Molecular markers genotyping

Cleaved Amplified Polymorphic Sequences (CAPS) markers were previously used for mapping in *A*. *bisporus* [29–30] and to study the life cycle of *A*. *subrufescens* [16, 26]. They exploit heteromorphic positions detected in the sequences. In this study, loci showing restriction fragment length polymorphism between alleles within strain CA487 were included for analyzing the SSIs. After digestion with the appropriate restriction endonuclease, DNA fragments were separated by electrophoresis in 2% agarose gel. Co-dominant alleles and their constituent DNA fragment sizes are given in Tables 1 and 2.

**Table 2:** ITS phenotypes of 94 single spore isolates of strain CA487 and their genotypic interpretation under the hypothesis of two loci *ITSI* and *ITSII*.

Types	Phenotypic classes			Genotypic classes among					
of	Lengths of DNA fragments^a^ after digestion by	N^b^		69 homokaryons^c^				25 heterokaryons^c^		
ITS	enzyme *Mbo*II	enzyme *Fok*I		*ITSI*	*ITSII*	N^b^	*ITSI*	*ITSII*	N^b^
[A]	396 (+377)	773	21	*a*	*n*^*d*^	19	*a*/*a*	*n/n*	2
[B]	772	563 (+209)	23	*b*	*n*	20	*b*/*b*	*n/n*	3
[C]	395+264 (+112)	562 (+209)	0			0			0
[A+C]	396+264 (+395+377+112)	773+562 (+209)	21	*a*	*c*	19	*a*/*a*	*c*/*n* or *c*/*c*	2
[A+B]	772+396 (+377)	773+563 (+209)	1			0	*a*/*b*	*n/n*	1
[B+C]	772+395+264 (+112)	563 (562+209)	17	*b*	*c*	11	*b*/*b*	*c/n* or *c*/*c*	6
[A+B+C]	772+396+264 (+395+377+112)	773+563 (562+209)	11			0	*a*/*b*	*c/n* or *c*/*c*	11

^a^ Smallest or redundant uninformative bands in electrophoretic patterns are in parentheses

^b^ N is the number of single spore isolates among each considered offspring

^c^ Rates of homokaryotic and heterokarotic offspring have been determined independently by using other markers

^d^ Null allele

### Multilocus genotype testing

A multilocus genotype test previously used in *A*. *bisporus* by Kerrigan et al. [31–33] and in *A*. *subrufescens* by Thongklang et al. [16] and Rocha de Brito et al. [26] to determine the homozygote/heterozygote status of the SSIs (n vs. n+n) was also used here. Specifically, co-dominant single-locus markers that are heteroallelic in the parental strain are used to determine whether the individual SSIs were homozygotes or heterozygotes. SSIs heteroallelic at least at one of the loci are unambiguously heterokaryons. SSIs homoallelic at all loci are regarded as putative homokaryons. This multilocus genotype test requires markers that must be genetically independent (genetically unlinked loci) and is more stringent if one of the markers is tightly linked to a centromere [16]. Contingency Chi-square tests (*df* = 1, *p* = 0.05) were used to determine the linkage relationships among the markers.

## Results

### Types of ITS inferred from 284 clones

Since double or triple peaks were observed in chromatograms by direct sequencing, PCR products of the heterokaryotic parental strain CA487 were cloned to reveal the ITS sequence diversity within the strain. The sequences obtained from 284 clones did not exhibit any double or triple peaks. In the ITS alignment of the 284 sequences, 35 of the 662 aligned nucleotide sites were variable.

Among the 284 clones, those having identical sequences were grouped in the same type; 164 clones were grouped into three major types that we named ITS types A, B, and C. Type A (59 clones) was characterized by having unique nucleotides at six positions (39, 122, 130, 145–146, and 200); type B (47 clones) by having unique nucleotides at four positions (114, 334, 559, and 649); and type C (58 clones) by having unique nucleotides at three positions (269, 466, 475). Together, alignment of the 164 cloned ITS sequences showed polymorphism at 13 positions (8 in ITS1, 1 in 5.8S, and 4 in ITS2) as indicated in Table 3. The remaining 120 sequences differed from the main ITS types A, B, or C either by nucleotide substitutions at single or rarely two unrelated positions or were putative chimeras due to recombination between the main ITS types. The A, B, and C type sequences are the “dominant” sequences and the focus of our study while others may represent true minor variants or PCR/sequencing artifacts. A more intense sequencing is needed to confirm whether the minor variants represent authentic variants or PCR artifacts. The ratio between the observed numbers of clones of types A, B, and C among the 164 sequenced clones (59, 47, and 58, respectively) did not differ significantly from a 1:1:1 ratio (Chi-square value = 1.62, *df* = 2, p = 0.44). Three representative sequences of each of ITS types A, B, and C sequences are deposited in GenBank (Table 3).

**Table 3:** Types of ITS revealed by polymorphisms at 13 positions among 164 clones of CA487.

Type	Polymorphic position													Number of clones
of ITS	39	114	122	130	145	146	200	269	334	466	475	559	649	(sequence type; GenBank #)
Isolate CA487														
ABC	T/–^a^	A/G	A/G	A/G	A/G	A/T	C/T	A/G	A/G	A/C	T/–	A/G	A/T	
164 clones having ITS of types A, B or C														
A	**T**	A	**A**	**A**	**G**	**A**	**T**	G	A	A	T	G	T	59 (CA487-C5; KU557352)
B	–	**G**	G	G	A	T	C	G	**G**	A	T	**A**	**A**	47 (CA487-C6; KU557353)
C	–	A	G	G	A	T	C	**A**	A	**C**	**–**	G	T	58 (CA487-C2; KU557351)

^a^ In this alignment, absent nucleotides are represented by a dash and characters specific to each type of ITS appear in bold type.

### Confirmation of the stable presence of three different ITS sequences within isolate CA487

In the original ITS sequence chromatograph of isolate CA487, we observed some heteromorphisms and triple peaks that could not be explained by superposing the ITS sequences of two homokaryotic single spore isolates CA487-S100 and CA487-S42 obtained by Thongklang et al. [16]. We hypothesized that a third ITS type might be present in isolate CA487. As explained in Fig 1, based on the original chromatograph of strain CA487 and the chromatographs of its two SSIs, it has been possible to reconstitute the three types of ITS with the exception of the character G at position 334 located in the 5.8S region. This G was masked by the flanking G of the sequences of types A or C in both the forward and reverse strand, respectively (Fig 1). This polymorphism was detected in another sequence obtained by using a forward primer located between the length heteromorphism of the position 39 and the position 334 (data not shown). It was also detected in the sequence of CA487-S42. These three sequences were confirmed by results from sequencing of cloned ITS PCR products. The ITS sequences of the single spore isolates CA487-S100 and CA487-S42 [16] were found to be identical to sequences of types A and B, respectively.

To further verify our results and eliminate the possibility of contamination, we also extracted DNA from additional tissues of the originally collected fruiting body (herbarium specimen CA487) as well as fruiting bodies produced by growing the mycelia of this specimen (isolate CA487). Our results showed that the presence of the three types of ITS was stable. In addition, we also sequenced the specimen CA516 collected in the same site about one month later. Chromatographs with similar double and triple peaks were obtained in all cases.

### ITS sequences comparison of selected samples from different continents

Representative clones (ITS of types A, B, and C) of isolate CA487 were compared with sequences of 18 wild specimens from Americas, Asia, Europe, and Oceania. Among three samples that were highly heterozygous for the ITS sequences, two isolates had clear haploid sequences of the constitutive nuclei and thus they were included in the comparison, while the remaining highly heterozygous sequence was excluded. From the total alignment, 29 positions were found variable; polymorphisms at these positions are shown in S1 Table. At nine of the 29 positions the polymorphisms are parsimony informative (i.e. with each nucleotide shared by at least two groups of samples) and allowed us to distinguish three groups of ITS, each being characterized by its own unique set of nucleotides (haplotype) at these 9 positions (Table 4). Specifically, two of the three groups of sequences are characterized by exactly the same six and three nucleotides which characterized respectively the types A and C of strain CA487 (S1 Table). The sequences of the third group have the same set of nucleotides (haplotype) than as the type B of CA487 at the nine polymorphic positions. However, the four nucleotides which characterized the type B sequence within isolate CA487 were not shared by any other sequences of our sample and thus appeared as non-parsimony informative. Other sequences of type A or C have also one or several polymorphisms at other position not shared by all sequences within each clade.

**Table 4:** Nucleotides at nine polymorphic positions in ITS sequences of 19 specimens of *A*. *subrufescens* from different geographic regions.

Sample	Geographic	GenBank	Ploidy	Type	Position^a^								
	origin	Number	level^b^	ITS	39	122	130	145	146	200	269	466	475
Samples or sequences with one type of ITS													
CA864-A	France		Ho (D)	A	**T**	**A**	**A**	**G**	**A**	**T**	G	A	T
CA487-C5	France	KU557352	Ho (C)	A	**T**	**A**	**A**	**G**	**A**	**T**	G	A	T
WC837-S43	Brazil	KJ541797	Ho (S)	A	**T**	**A**	**A**	**G**	**A**	**T**	G	A	T
F2285	Martinique	JF797201	He	A/A	**T**	**A**	**A**	**G**	**A**	**T**	G	A	T
CA864-B	France		Ho (D)	B	–	G	G	A	T	C	G	A	T
CA487-C6	France	KU557353	Ho (C)	B	–	G	G	A	T	C	G	A	T
WC837-S04	Brazil	KJ541796	Ho (S)	B	–	G	G	A	T	C	G	A	T
L0341732	UK	AY818649	He	B/B	–	G	G	A	T	C	G	A	T
CA487-C2	France	KU557351	Ho (C)	C	–	G	G	A	T	C	**A**	**C**	**–**
GY121118	China	KJ755633	He	C/C	–	G	G	A	T	C	**A**	**C**	**–**
GY128956	China	KJ755632	He	C/C	–	G	G	A	T	C	**A**	**C**	**–**
GY128883	China	KJ755634	He	C/C	–	G	G	A	T	C	**A**	**C**	**–**
GY133048	China	KJ755635	He	C/C	–	G	G	A	T	C	**A**	**C**	**–**
XHW1614	China	KJ755636	He	C/C	–	G	G	A	T	C	**A**	**C**	**–**
CA918	Thailand	KJ541798	He	C/C		G	G	A	T	C	**A**	**C**	**–**
ZRL2036	Thailand	KU557345	He	C/C	–	G	G	A	T	C	**A**	**C**	**–**
ZRL2134	Thailand	KU557346	He	C/C	–	G	G	A	T	C	**A**	**C**	**–**
NT001	Thailand	KU557347	He	C/C	–	G	G	A	T	C	**A**	**C**	**–**
CA935	Thailand	KU557348	He	C/C	–	G	G	A	T	C	**A**	**C**	**–**
KRP070	Hawaii	AY818648	He	C/C	–	G	G	A	T	C	**A**	**C**	**–**
DEH513	Hawaii	AY818646	He	C/C	–	G	G	A	T	C	**A**	**C**	**–**
DEH1073	Hawaii	AY818647	He	C/C	–	G	G	A	T	C	**A**	**C**	**–**
Samples possessing two or three different types of ITS^c^													
WC837	Brazil	KU557350	He	A/B	T/–	A/G	A/G	**G**/A	**A**/T	**T**/C	G	A	T
SBRF	USA-CA	AY818657	He	(A/B)^d^	T/–	A/G	A/G	**G**/A	**A**/T	**T**/C	G	A	T
CA864	France	KU557349	He	A/B	T/–	A/G	A/G	**G**/A	**A**/T	**T**/C	G	A	T
CA487	France		He	A/B/C	T/–	A/G	A/G	**G**/A	**A**/T	**T**/C	G/**A**/	A/**C**	T/**–**

^a^ Absent nucleotides are indicated as deletion by a dash; ITS type A or type C-specific characters are in bold type.

^b^ He = heterokaryotic genotype (*n+n*); Ho = homokaryotic genotype (*n*) were obtained from clone (C), from spore (S), or deduced (D) from the electropherogram of a heterokaryon by interpreting the double peaks.

^c^ Heterokaryotic specimens possessing several types of ITS; their haploid constituents A, B, or C, are listed above in the Table, when known.

^d^ Presumably A/B since SBRF has the same characteristic polymorphisms than WC837 at nine positions

Several samples from Europe and Americas such as the French strain CA864 and the Brazilian strain WC837 that were highly heterozygous possessed in fact both haploid sequences of type A and B (Table 4). This is also likely the case for the Californian sample SBRF, but this was not formally shown because haploid sequences were not available. For this reason, the sequence of SBRF was not included in the phylogenetic analysis.

**Fig 2:**
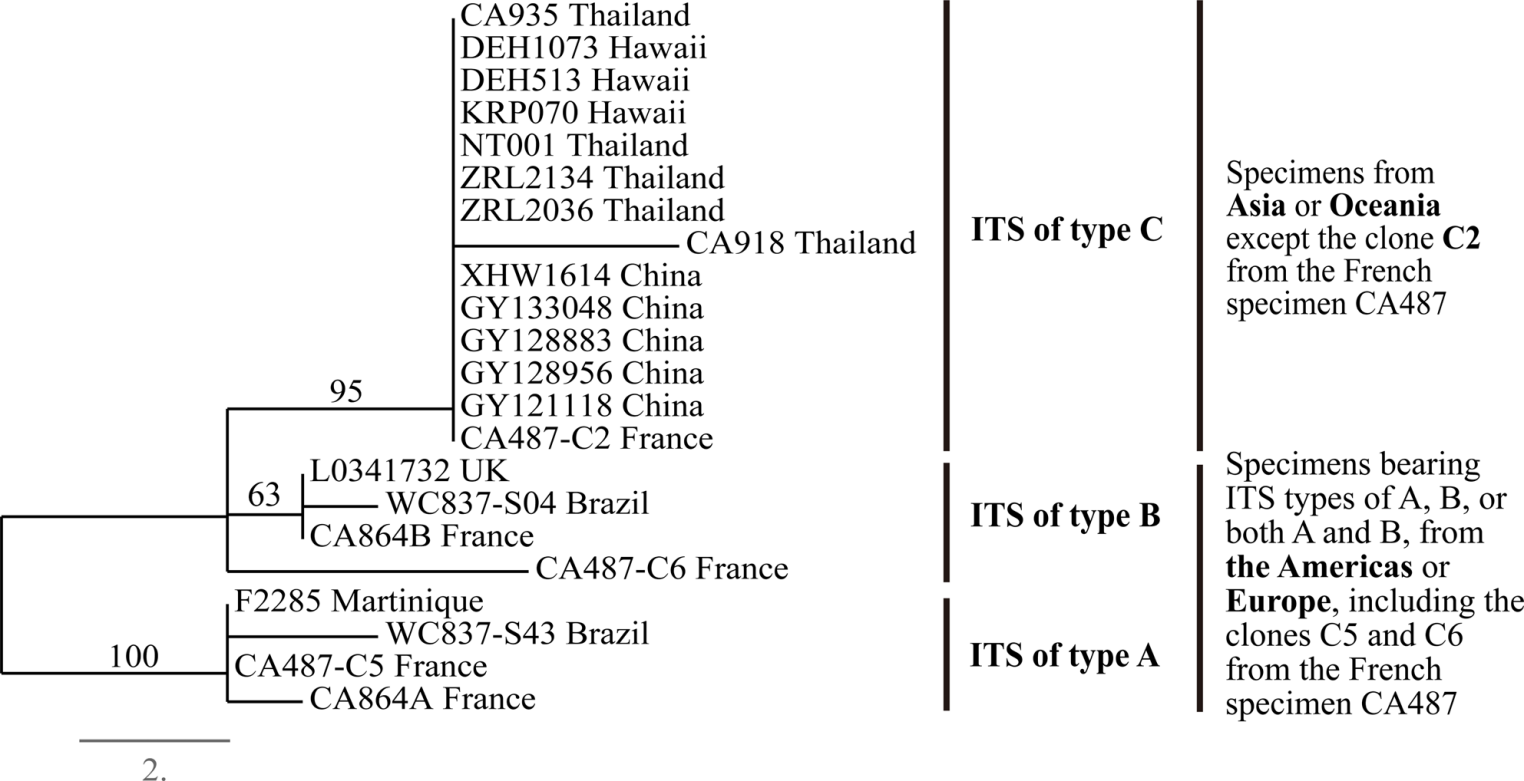
One of four equally parsimonious trees drawn from 22 ITS sequences of *Agaricus subrufescens* from different geographic regions. The bootstrap support values greater than 50% are indicated. ITS of type A is characterized by six alleles: *ITS39-T*, *ITS22-A*, *ITS130-A*, *ITS145-G*, *ITS146-A* and *ITS200-T*; ITS of type B is not characterized by any specific alleles; ITS of type C is characterized by three alleles: *ITS269-A*, *ITS466-C* and *ITS475-deletion*.

The relationships among the 22 ITS sequences are shown in Fig 2. The results indicated the types A and C ITS sequences belonged to two distinct lineages with strong bootstrap support, while the sequences of type B form a paraphyletic group. Geographically, specimens with ITS types A, B or both A and B were found in the Americas and in Europe, while sequences of type C were found in specimens from Hawaii and Southeast Asia. The only exception was the type C sequence from the French isolate CA487 which also possessed the types A and B sequences.

### Classification of the single spore isolates: homokaryons and heterokaryons

The heterozygosity level of SSIs (*n* vs. *n+n*) was determined on the basis of the homo- or hetero-allelic genotypes at three genetically independent loci, obtained by using three CAPS markers (Table 1 and S2 Table). Among the 94 SSIs, 26.60% (25/94) were heteroallelic at least at one of the three loci and were therefore considered as heterokaryons. The remaining SSIs were putative homokaryons and further subjected to ITS genotyping with PCR-RFLP. From Chi-square contingency tests, Mendelian segregation 1:1 was not rejected at each locus and no evidence of genetic linkage was found among the three loci (*df* = 1, p > 0.05 in all tests, see S3 Table). We also noted that, among the 25 heterokayons, the rates of homozygosity were 60% (15/25), 56% (14/25), and 40% (10/25) at three loci *PRS49*, *PRS16*, and *PRS88* respectively, with an overall mean of 52%. As the locus *PRS88* is tightly linked to the *MAT* locus, the rate of homozygosity at this locus of 40% is also an estimation of the rate of sister nuclei heterokaryons that are homozygous at the *MAT* locus and behavior as homokaryons [26]. This observed rate is close to the 33% expected in a theoretical model of random migration of the post meiotic nuclei in the spores [26].

### ITS phenotypes and genotypes of the SSIs

The three ITS sequence types of A, B, and C can theoretically generate seven different combinations in offspring: A, B, C, A+B, A+C, B+C, and A+B+C. Among these seven possible combinations, only the pure type C was not found among the 94 SSI progeny. The restriction enzyme *Mbo*II has one recognition site present in both types A and C and a second recognition site present in only ITS type C (Table 1). However similar electrophoretic patterns are expected in two cases: for [C] and [A+C] and also for [B+C] and [A+B+C]. To ensure further distinction of the types, digestion using an additional enzyme *Fok*I was employed and this enzyme has a recognition site present in both types B and C. Together, digestions using *Mbo*II and *Fok*I distinguished all the phenotypes. As shown in Table 2, except the pure type C, all six other phenotypes were detected among the 94 single spore isolates. The number of each ITS phenotype and their putative genotypes are reported in Table 2.

Among the 69 putative homokaryons, phenotypes [C], [AB] and [A+B+C] were not found while the remaining four types [A], [B], [A+C], and [B+C] were found. The results are consistent with the presence of two independently segregating ITS loci *ITSI* and *ITSII* in isolate CA487. Specifically, ITS of types A (19 + 19 = 38) and B (20 + 11 = 31) bearing alleles *ITSI-a* and *ITSI-b*, respectively, segregate at locus *ITSI*. Homokaryons possessing the ITS type C (19 + 11 = 30) bear an allele *ITSII-c* segregating at the second locus *ITSII* with a corresponding null allele (*ITSII-n* as null in 39 homokaryons). Alleles at both loci *ITSI* and *ITSII* showed a balanced Mendelian segregation (Chi-square values were of 0.72 and 1.18, respectively, which means 1:1 segregation ratio is accepted; *df* = 1, p > 0.05). For the Chi-square contingency tests of genetic linkage between the two loci *ITSI* and *ITSII*, the parental: recombinant ratio was 30:39 (Chi-square value = 1.43, *df* = 1, p = 0.23), not significantly different from independent re-assortment. Similar results were obtained for *ITSI*, *ITSII* and the three markers used in this study (S3 Table) showing that alleles at all five loci were segregating normally and re-assorting independently.

Among the genotypes of the 25 heterokaryons at the *ITSI* locus, the observed pairing of the alleles in the spores is 4 *a/a*, 9 *b/b*, and 12 *a/b*, giving a homozygosity of 52% (13/25) which is similar to the rate observed on average at the three other loci *PRS49*, *PRS16*, *PRS88* in the same offspring. This is also similar to the rate around 50% observed at various loci in different offspring by Thongklang et al. [16] or Rocha de Brito et al. [26]. At the *ITSII* locus genotypes *ITSII-c/c* and *ITSII-c/n* have the same phenotype [C] and thus cannot be distinguished from each other. At this locus, the observed homozygosity at *ITSII* for the null allele was 24% (6/25), which is also in agreement with a rate around 50% of homozygosity at this locus for the two alleles.

In conclusion, allelic segregation among both homokaryotic and heterokaryotic offspring agree with the presence of two unlinked ITS loci. ITS of types A and B are two alleles segregating at the *ITSI* locus. ITS of type C is a non-allelic homolog (a paralog) located at the second locus *ITSII* that is not linked to *ITSI*.

## Discussion

### Evidence for the presence of three different types of ITS

The presence of three distinct types of ITS within one strain was supported by data from different methods. First they were inferred by interpreting the shifted sequences in the chromatograms of the isolate CA487 due to length differences among the three types. Similar chromatograms were obtained with CA516, a collection from the same site, indicating that a mycelium possessing these three ITS sequences was persisting at this location. Among the hundreds of ITS sequences of specimens in the genus *Agaricus* that we have obtained since over a decade ago, this was the first time we observed such stable and reproducible chromatograms with triple peaks. Then the presence of three distinct ITS sequence types was confirmed by the more reliable method of PCR cloning and sequencing. The observed number of clones bearing three types of ITS (A, B, and C) was not significantly different from the 1:1:1 ratio in our PCR clone library. The three types of ITS were also found in the offspring but none of 94 single spore isolates possessed only the type C.

### Genetic hypotheses inferred from the analyses of the offspring

Among the 94 single spore isolates, 25 (27%) showed evidence of heterozygosity and were considered heterokaryotic. This is lower than the 44% estimated previously among a different set of offspring of the same strain by Thongklang et al. [16]. However, the previously estimated rate was likely an overestimate because their ITS marker could not distinguish ITS types B and C and homokaryons [AC] were interpreted as heterokaryons. In addition, variations in environmental conditions might influence the rate of heterokarotic offspring in both amphithallic species *A*. *subrufescens* and *A*. *bisporus* [31].

The presence of the different types of ITS and their segregation were separately analyzed in the homokaryotic and the heterokaryotic offspring. Based on the fact that the three types of ITS were equally prevalent in the parent strain, one simple hypothesis was that CA487 was a trikaryon, i.e. a heterokaryon composed of three types of genetically different nuclei [34] with each of them carrying the ITS of types A, B and C, respectively. The presence of homokaryons AC and BC, and the absence of homokaryons C in the homokaryotic offspring allow us to definitely reject this hypothesis. Instead, we observed allelic segregations among both homokaryotic and heterokaryotic offspring which agree with the presence of two unlinked loci. ITS of types A and B are two alleles at the *ITSI* locus. ITS of type C segregated with a null allele at the *ITSII* locus unlinked to *ITSI*. At *ITSI*, the rate of homozygosity was close to 50%, similar to the other loci. *ITSI* and *ITSII* are not linked to any other marker used in this study.

The locus *ITSI* may contain hundreds of copies of the ITS and here we assume the simplest explanation that all the copies of the allele *ITSI-a* were of type A and all the copies of the allele *ITS-b* were of type B. The hypothesis that a proportion of the copies of the allele *ITS-a* could be of type B sequence is possible but unlikely for two reasons. Firstly, the chromatograms of type A and type B sequences in types A and B homokaryons were very clean with no obvious minor peaks resulting from the differences between the two sequences. Secondly, wild strains with only type A or type B are found in Europe, North and South Americas and the Caribbean. Therefore, alleles A and B seemed to have diverged a long time ago. If types A and B sequences could be mixed in the same cluster within the same homokaryon, we should expect to find all the intermediate proportions between 100% A 0% B and 0% A 100% B. However, in the different offspring we have studied (present and previous studies), we have never found any homokaryon with both the type A and type B sequences.

The heterokaryon CA487 has two constituent nuclei bearing alleles *ITSI-a* and *ITSI-b*, respectively, but it remains unknown in which of the two nuclei the *ITSII* locus can be found. Unfortunately, attempts to de-heterokaryotize CA487 to obtain the two constitutive haploid nuclei have failed. The methods we used were the same protocols that have allowed us to successfully obtain homokaryons from heterokaryons in *A*. *bisporus* through protoplasting.

Our data indicated that *ITSI* is not linked to the centromere. Specifically, the heterokaryons that are homoallelic at the centromere-linked locus *PRS088* are putative sister progeny heterokaryons and thus should be highly homozygous at all centromere-linked loci [26]. This is not the case at the *ITSI* locus since four of these ten heterokaryons are heterozygous *ITSI-a/b*. As for the second ITS locus, it is possible that the null allele represents undetected ITS that could not be amplified with the current universal primers. However, we believe this is highly unlikely as we have not failed amplification of any samples from the genus *Agaricus* using the universal primers. The simplest hypothesis is that *ITSII* is a duplicate locus of the ITS introgressed from a homokaryon closely related to those from an Asian strain and that it is present in only one of the two constitutive haploid nuclei of the parent CA487. *ITSII* is not linked to *ITSI* and this genomic composition is persisting in the natural environment.

### Geographic distribution ranges of the types of ITS and evidence for hybridization

ITS sequences similar to or identical with the types A, B, and C sequences of isolate CA487 have been found in field specimens from different continents. The extensive differences among the three sequences suggest that these sequences have likely diverged from each other for a long time. However, the exact reason for their divergence is not known. These divergences may have occurred several millions years ago. Indeed, an approximate date of 2–3 Ma is indicated in the multigene Maximum Clade Credibility tree of Zhao et al. [35] for the divergence between a wild Chinese sample (zrl2012722) and a cultivar (CA276). The latter likely originates from Brazil, like most cultivars having a ITS sequence of type A/B. In addition, *A*. *subrufescens* is the earliest species arising in the *A*. sect. *Arvenses* approximately 12 Ma ago. The geographical distribution of the different types of sequences suggests that geographical isolation might have contributed to their divergence.

Kerrigan [13] analyzed the diversity of *A*. *subrufescens* and noted extensive heterozygosity in American samples. He suggested that hybridization might have occurred between populations. He also noted that the Hawaiian samples were highly diverged from samples in the Americas. Since 2005, many specimens have been collected in Europe and in Asia. Some European samples possessed similar heteromorphisms as the American samples while ITS sequences of the Chinese and Thai samples were similar or even identical to those of the Hawaiian samples [13, 16, 21, 22]. Our analysis showed that American and European samples had ITS of type A, B or both A and B, while the Asian and Oceanian samples had ITS of type C. Thus, we were surprised to find an ITS sequence of type C in the French specimen CA487.

Our results suggest two possibilities. In the first, the type C sequence might have a distribution much broader than is currently known to cover Eurasia and even Africa. Incomplete sequences of old herbarium samples of *A*. *subrufescens* from DR Congo in Africa [17] showed a high sequence identity to type C sequences, suggesting that the type C might be present on this continent. Additional samples from Europe and Africa might help shed light on its distributions. In the second, the ITS of type C was recently introduced and introgressed from Asia/Oceania to Europe. The commercial cultivation of *A*. *subrufescens* remains sporadic in Europe and to our knowledge all or almost all the cultivated strains originated from America and most of them bear both types of ITS A and B sequences. Alternatively, human assisted migration of the three types of ITS sequences into the same ecological niche could have brought the different types together and resulted in their hybridization and subsequent generation of a strain such as CA487. We would like to note that CA487 was isolated in 2006, before *A*. *subrufescens* were formally described based on field specimens in Asia. In addition, to our knowledge, *A*. *subrufescens* was not commercially cultivated in Europe, and there was only few imports from Brazil [14].

Interestingly, since 2012, the Thai isolate CA918 possessing an ITS of type C has been cultivated and crossed with the Brazilian strain WC837 and the French strain CA487. These crosses generated strains with different combinations of ITS types such as AC or BC and these strains have been cultivated at INRA [16]. These observations showed that a Asian sample possessing the ITS of type C was inter-fertile with those bearing types A or B from Europe or Americas, giving viable and sometimes vigorous hybrids. Thus, it is conceivable a hybrid with all three ITS sequence types could be generated.

### Are ITS loci I and II within functional rDNA clusters?

In strain WC837 of *A*. *subrufescens*, Rocha de Brito et al. [25] found that the ITS locus was linked to a locus PRS160 located in a sequence having a homolog in a distal position on chromosome VII of *A*. *bisporus*. This result indicated that the position of *PR160* and/or of the *ITS* locus likely differed between these two species, despite their high level of macro-synteny [28]. In strain CA487, *ITSI* is most likely functional because offspring having only the *ITSI-a* or the *ITSI-b* alleles are viable. In contrast, the *ITSII-c* allele was never found alone in the single spore isolates, thus whether it is functional or not remains to be determined. Based on the chromatograms and the relative proportions of clones with the types A, B, and C sequences from strain CA487, the copy number of the type C ITS sequence within strain CA487 is likely very similar to those of types A and B. The long-term effects of such introgression and ITS duplication on the genome structure and function of *A*. *subrufescens* remain to be examined.

## Supporting Information

S1 Table Nucleotides at 29 polymorphic positions for 22 sequences of 18 specimens from different countries. (DOCX)

S2 Table Genotypes of 94 single spore isolates at five loci. (DOCX)

S3 Table Evidence for Mendelian segregation between alleles within each of the five loci and independent re-assortment between all pairs of loci. (DOCX)

## References

[1] Schoch CL, Seifert KA, Huhndorf S, Robert V, Spouge JL, Levesque CA, et al Nuclear ribosomal internal transcribed spacer (ITS) region as a universal DNA barcode marker for Fungi. Proc Natl Acad Sci USA. 2012;109(16): 6241–6246. 10.1073/pnas.1117018109 22454494 PMC3341068

[2] Ganley AR, Kobayashi T. Highly efficient concerted evolution in the ribosomal DNA repeats: total rDNA repeat variation revealed by whole-genome shotgun sequence data. Genome Res. 2007;17(2):184–191. 10.1101/gr.5457707.1 17200233 PMC1781350

[3] Smith ME, Douhan GW, Rizzo DM. Intra-specific and intra-sporocarp ITS variation of ectomycorrhizal fungi as assessed by rDNA sequencing of sporocarps and pooled ectomycorrhizal roots from a Quercus woodland. Mycorrhiza. 2007;18(1):15–22. 10.1007/s00572-007-0148-z 17710446

[4] Lindner DL, Banik MT. Intragenomic variation in the ITS rDNA region obscures phylogenetic relationships and inflates estimates of operational taxonomic units in genus *Laetiporus*. Mycologia. 2011;103(4):731–740. 10.3852/10-331 21289107

[5] Li Y, Jiao L, Yao YJ. Non-concerted ITS evolution in fungi, as revealed from the important medicinal fungus *Ophiocordyceps sinensis*. Mol Phylogenet Evol. 2013;68(2):373–379. 10.1016/j.ympev.2013.04.010 23618625

[6] Hillis DM, Moritz C, Porter CA, Baker RJ. Evidence for biased gene conversion in concerted evolution of ribosomal DNA. Science. 1991;251(4991):308 10.1126/science.1987647 1987647

[7] Coen ES, Dover GA. Unequal exchanges and the coevolution of X and Y rDNA arrays in *Drosophila melanogaster*. Cell. 1983;33(3):849–855. 10.1016/0092-8674(83)90027-2 6409420

[8] Weitemier K, Straub SC, Fishbein M, Liston A. Intragenomic polymorphisms among high-copy loci: a genus-wide study of nuclear ribosomal DNA in *Asclepias* (Apocynaceae). PeerJ. 2015;3:e718 10.7717/peerj.718 25653903 PMC4304868

[9] O'Donnell K, Cigelnik E. Two divergent intragenomic rDNA ITS2 types within a monophyletic lineage of the fungus *Fusarium* are nonorthologous. Mol Phylogenet Evol. 1997;7(1):103–116. 10.1006/mpev.1996.0376 9007025

[10] Hijri M, Hosny M, van Tuinen D, Dulieu H. Intraspecific ITS polymorphism in *Scutellospora castanea* (Glomales, Zygomycota) is structured within multinucleate spores. Fungal Genet Biol. 1999;26(2):141–151. 10.1006/fgbi.1998.1112 10328984

[11] Wang DM, Yao YJ. Intrastrain internal transcribed spacer heterogeneity in *Ganoderma* species. Can J Microbiol. 2005;51(2):113–121. 10.1139/W04-118 16091769

[12] Fell JW, Scorzetti G, Statzell-Tallman A, Boundy-Mills K. Molecular diversity and intragenomic variability in the yeast genus *Xanthophyllomyces*: the origin of *Phaffia rhodozyma*?. FEMS Yeast Res. 2007;7(8):1399–1408. 10.1111/j.1567-1364.2007.00297.x 17825066

[13] Kerrigan RW. *Agaricus subrufescens*, a cultivated edible and medicinal mushroom, and its synonyms. Mycologia. 2005;97(1):12–24. 10.3852/mycologia.97.1.12 16389952

[14] Largeteau ML, Llarena-Hernández RC, Regnault-Roger C, Savoie JM. The medicinal *Agaricus* mushroom cultivated in Brazil: biology, cultivation and non-medicinal valorisation. Appl Microbiol Biotechnol. 2011;92(5):897–907. 10.1007/s00253-011-3630-7 22005742

[15] Wisitrassameewong K, Karunarathna SC, Thongklang N, Zhao RL, Callac P, Moukha S, et al *Agaricus subrufescens*: a review. Saudi J Biol Sci. 2012;19(2):131–146. 10.1016/j.sjbs.2012.01.003 23961172 PMC3730566

[16] Thongklang N, Hoang E, Estrada AE, Sysouphanthong P, Moinard M, Hyde KD, et al Evidence for amphithallism and broad geographical hybridization potential among *Agaricus subrufescens* isolates from Brazil, France, and Thailand. Fungal Biol. 2014;118(12):1013–1023. 10.1016/j.funbio.2014.10.004 25457949

[17] Thongklang N, Chen J, Bandara AR, Hyde KD, Raspé O, Parra LA, et al Studies on *Agaricus subtilipes*, a new cultivatable species from Thailand, incidentally reveal the presence of *Agaricus subrufescens* in Africa. Mycoscience. 2016; in press. 10.1016/j.myc.2016.02.003

[18] Chen J, Parra LA, De Kesel A, Khalid AN, Qasim T, Ashraf A, et al Inter-and intra-specific diversity in *Agaricus endoxanthus* and allied species reveals a new taxon, *A*. *punjabensis*. Phytotaxa. 2016;252(1):1–16. 10.11646/phytotaxa.252.1.1

[19] Rees BJ, Midgley DJ, Marchant A, Perkins A, Orlovich DA. Morphological and molecular data for Australian *Hebeloma* species do not support the generic status of *Anamika*. Mycologia. 2013;105(4):1043–1058. 10.3852/12-404 23709478

[20] Llarena-Hernández CR, Largeteau ML, Ferrer N, Regnault-Roger C, Savoie JM. Optimization of the cultivation conditions for mushroom production with European wild strains of *Agaricus subrufescens* and Brazilian cultivars. J Sci Food Agric. 2014;94(1):77–84. 10.1002/jsfa.6200 23633302

[21] Zhao RL, Karunarathna S, Raspé O, Parra LA, Guinberteau J, Moinard M, et al Major clades in tropical *Agaricus*. Fungal Divers. 2011;51(1):279–296. 10.1007/s13225-011-0136-7

[22] Gui Y, Zhu GS, Callac P, Hyde KD, Parra LA, Chen J, et al *Agaricus* section *Arvenses*: three new species in highland subtropical Southwest China. Fungal Biol. 2015;119(2):79–94. 10.1016/j.funbio.2014.10.005 25749361

[23] Wisitrassameewong K, Karunarathna SC, Thongklang N, Zhao RL, Callac P, Chukeatirote E, et al *Agaricus subrufescens*: new to Thailand. Chiang Mai J Sci. 2012;39(2):281–291. 10.1016/j.sjbs.2012.01.003 PMC3730566 23961172

[24] Hall TA. BioEdit: a user-friendly biological sequence alignment editor and analysis program for Windows 95/98/NT. Nucleic Acids Symp Ser. 1999;41:95–98.

[25] Swofford DL. PAUP*. Phylogenetic analysis using parsimony (and other methods) Version 4. 2002; Sinauer Associates, Sunderland, MA.

[26] Rocha de Brito M, Foulongne-Oriol M, Moinard M, Dias ES, Savoie JM, Callac P. Spore behaviors reveal a category of mating-competent infertile heterokaryons in the offspring of the medicinal fungus *Agaricus subrufescens*. Appl Microbiol Biotechnol. 2016;100(2):781–796. 10.1007/s00253-015-7070-7 26497018

[27] Foulongne-Oriol M, Lapalu N, Férandon C, Spataro C, Ferrer N, Amselem J, et al The first set of expressed sequence tags (EST) from the medicinal mushroom *Agaricus subrufescens* delivers resource for gene discovery and marker development. Appl Microbiol Biotechnol. 2014;98(18):7879–7892. 10.1007/s00253-014-5844-y 24917377

[28] Foulongne-Oriol M, de Brito MR, Cabannes D, Clément A, Moinard M, Spataro C, et al The Genetic Linkage Map of the Medicinal Mushroom *Agaricus subrufescens* Reveals Highly Conserved Macrosynteny with the Congeneric Species *Agaricus bisporus*. G3 Genes Genom Genet. 2016 10.1534/g3.115.025718 PMC4856074 26921302

[29] Callac P, Desmerger C, Kerrigan RW, Imbernon M. Conservation of genetic linkage with map expansion in distantly related crosses of *Agaricus bisporus*. FEMS Microbiol Lett. 1997;146(2):235–240. 10.1016/S0378-1097(96)00482-X 9011044

[30] Foulongne-Oriol M, Spataro C, Cathalot V, Monllor S, Savoie JM. An expanded genetic linkage map of *Agaricus bisporus* based on AFLP, SSR and CAPS markers sheds light on the recombination behavior of the species. Fungal Genet Biol. 2010;47(3):226–236. 10.1016/j.fgb.2009.12.003 20026415

[31] Kerrigan RW, Baller LM, Horgen PA, Anderson JB. Strategies for the efficient recovery of *Agaricus bisporus* homokaryons. Mycologia. 1992;84(4):575–579. 10.2307/3760324

[32] Kerrigan RW. New prospects for *Agaricus bisporus* strain improvement. Reports of the Tottori Mycological Institute (Japan). 1993;31:188–200.

[33] Kerrigan RW, Imbernon M, Callac P, Billette C, Olivier JM. The heterothallic life cycle of *Agaricus bisporus* var. *burnettii* and the inheritance of its tetrasporic trait. Exp Mycol. 1994;18(3):193–210. 10.1006/emyc.1994.1020

[34] James TY, Johansson SB, Johannesson H. Trikaryon formation and nuclear selection in pairings between heterokaryons and homokaryons of the root rot pathogen *Heterobasidion parviporum*. Mycol Res. 2009;113(5):583–90. 10.1016/j.mycres.2009.01.006 19640398

[35] Zhao RL, Zhou JL, Chen J, Margaritescu S, Sánchez-Ramírez S, Hyde KD, et al Towards standardizing taxonomic ranks using divergence times–a case study for reconstruction of the *Agaricus* taxonomic system. Fungal Divers. 2016.

