# Correction: Genetic Analyses of the Internal Transcribed Spacer Sequences Suggest Introgression and Duplication in the Medicinal Mushroom *Agaricus subrufescens*

**DOI:** 10.1371/journal.pone.0157927

**Published:** 2016-07-08

**Authors:** Jie Chen, Magalie Moinard, Jianping Xu, Shouxian Wang, Marie Foulongne-Oriol, Ruilin Zhao, Kevin D. Hyde, Philippe Callac

Table 2 appears incorrectly in the published article. Please see the correct Table 2 and its caption here.

**Table 2:** ITS phenotypes of 94 single spore isolates of strain CA487 and their genotypic interpretation under the hypothesis of two loci *ITSI* and *ITSII*.

Types Of ITS	Phenotypic classes			Genotypic classes among					
	Lengths of DNA fragments^a^ after digestion by		N^b^	69 homokaryons^c^			25 heterokaryons^c^		
	enzyme *Mbo*II	enzyme *Fok*I		*ITSI*	*ITSII*	N^b^	*ITSI*	*ITSII*	N^b^
[A]	396 (+377)	773	21	*a*	*n*^d^	19	*a*/*a*	*n/n*	2
[B]	772	563 (+209)	23	*b*	*n*	20	*b*/*b*	*n/n*	3
[C]	395+264 (+112)	562 (+209)	0			0			0
[A+C]	396+264 (+395+377+112)	773+562 (+209)	21	*a*	*c*	19	*a*/*a*	*c*/*n* or *c*/*c*	2
[A+B]	772+396 (+377)	773+563 (+209)	1			0	*a*/*b*	*n/n*	1
[B+C]	772+395+264 (+112)	563 (562+209)	17	*b*	*c*	11	*b*/*b*	*c/n* or *c*/*c*	6
[A+B+C]	772+396+264 (+395+377+112)	773+563 (562+209)	11			0	*a*/*b*	*c/n* or *c*/*c*	11

^a^ Smallest or redundant uninformative bands in electrophoretic patterns are in parentheses

^b^ N is the number of single spore isolates among each considered offspring

^c^ Rates of homokaryotic and heterokarotic offspring have been determined independently by using other markers

^d^ Null allele
