# Supplementary material for: Genetic Analyses of the Internal Transcribed Spacer Sequences Suggest Introgression and Duplication in the Medicinal Mushroom Agaricus subrufescens
Source: PLoS One. 2016 May 26;11(5):e0156250. doi: 10.1371/journal.pone.0156250 (PMC4882077; doi:10.1371/journal.pone.0156250)
Supplement: S1 Table — (DOCX) [file pone.0156250.s001.docx]

| Cou^a^ | Sample^b^ | Position | |  |  |  |  |  |  |  |  |  |  |  |  |  |  |  |  |  |  |  |  |  |  |  |  |  |  |  |
| --- | --- | --- | --- | --- | --- | --- | --- | --- | --- | --- | --- | --- | --- | --- | --- | --- | --- | --- | --- | --- | --- | --- | --- | --- | --- | --- | --- | --- | --- | --- |
|  |  | **39** | 46 | 53 | 97 | 114 | **122** | 123 | 126 | **130** | **145** | **146** | 154 | 168 | 190 | **200** | 233 | **269** | 334 | **466** | **475** | 476 | 478 | 493 | 531 | 541 | 559 | 481 | 589 | 649 |
| Sequences of type A | | |  |  |  |  |  |  |  |  |  |  |  |  |  |  |  |  |  |  |  |  |  |  |  |  |  |  |  |  |
| Fra | CA487-C5 | **T** | T | G | T | A | **A** | T | T | **A** | **G** | **A** | C | G | C | **T** | T | G | A | A | T | G | A | C | T | A | G | T | A | T |
| Fra | CA864-A | **T** | T | G | T | A | **A** | T | T | **A** | **G** | **A** | C | G | C | **T** | T | G | A | A | T | G | A | T | T | A | G | T | A | T |
| Bra | WC837-S43 | **T** | T | G | T | A | **A** | T | T | **A** | **G** | **A** | C | A | T | **T** | T | G | A | A | T | G | A | C | T | A | G | T | A | T |
| Mar | F2285 | **T** | T | R | T | A | **A** | T | T | **A** | **G** | **A** | C | G | C | **T** | T | G | A | A | T | G | A | Y | T | A | G | T | A | T |
| Sequences of type B | | |  |  |  |  |  |  |  |  |  |  |  |  |  |  |  |  |  |  |  |  |  |  |  |  |  |  |  |  |
| Fra | CA487-C6 | - | T | G | T | G | G | T | T | G | A | T | C | G | C | C | T | G | G | A | T | G | A | C | T | A | A | T | A | A |
| Fra | CA864-B | - | T | G | T | A | G | T | T | G | A | T | C | G | C | C | T | G | A | A | T | G | A | C | T | A | G | T | G | T |
| Bra | WC837-S04 | - | T | G | T | A | G | T | T | G | A | T | T | G | C | C | T | G | A | A | T | G | A | C | T | A | G | T | G | T |
| UK | L0341732 | - | T | G | T | A | G | T | T | G | A | T | C | G | C | C | T | G | A | A | T | G | A | C | T | A | G | T | G | T |
| Sequences of type C | | |  |  |  |  |  |  |  |  |  |  |  |  |  |  |  |  |  |  |  |  |  |  |  |  |  |  |  |  |
| Fra | CA487-C2 | - | T | G | T | A | G | T | T | G | A | T | C | G | C | C | T | **A** | A | **C** | **-** | G | A | C | T | A | G | T | A | T |
| Chi | GY121118 | - | T | G | T | A | G | T | T | G | A | T | C | G | C | C | T | **A** | A | **C** | **-** | K | A | C | T | A | G | T | A | T |
| Chi | GY128956 | - | T | G | T | A | G | T | T | G | A | T | C | G | C | C | T | **A** | A | **C** | **-** | G | A | C | T | M | G | T | A | T |
| Chi | GY128883 | - | T | G | T | A | G | T | T | G | A | T | C | G | C | C | T | **A** | A | **C** | **-** | G | A | C | T | A | G | T | A | T |
| Chi | GY133048 | - | T | G | T | A | G | T | T | G | A | T | C | G | C | C | T | **A** | A | **C** | **-** | G | A | C | T | A | G | T | A | T |
| Chi | XHW1614 | - | T | G | T | A | G | T | T | G | A | T | C | G | C | C | T | **A** | A | **C** | **-** | G | A | C | T | A | G | T | A | T |
| Tha | CA918 | - | Y | G | T | A | G | C | C | G | A | T | C | G | C | C | C | **A** | A | **C** | **-** | G | R | C | T | A | G | T | A | T |
| Tha | ZRL2036 | - | T | G | T | A | G | T | T | G | A | T | C | G | C | C | T | **A** | A | **C** | **-** | G | A | C | Y | A | G | Y | A | T |
| Tha | ZRL2134 | - | T | G | T | A | G | T | T | G | A | T | C | G | C | C | T | **A** | A | **C** | **-** | G | A | C | T | A | G | T | A | T |
| Tha | NT001 | - | T | G | W | A | G | Y | Y | G | A | T | C | G | C | C | T | **A** | A | **C** | **-** | G | A | C | T | A | G | T | A | T |
| Tha | CA935 | - | T | G | T | A | G | T | T | G | A | T | C | G | C | C | T | **A** | A | **C** | **-** | G | A | C | T | A | G | T | A | T |
| Haw | KRP070 | - | T | G | T | A | G | T | T | G | A | T | C | G | C | C | T | **A** | A | **C** | **-** | G | A | C | T | A | G | T | A | T |
| Haw | DEH513 | - | T | G | T | A | G | T | T | G | A | T | C | G | C | C | T | **A** | A | **C** | **-** | G | A | C | T | A | G | T | A | T |
| Haw | DEH1073 | - | T | G | T | A | G | T | T | G | A | T | C | G | C | C | T | **A** | A | **C** | **-** | G | A | C | T | A | G | T | A | T |

^a^ Countries: Fra: France, Bra: Brazil, Chi: Chine, Tha: Thailand, Haw: Hawaii

^b^ CA487-C5, CA487-C6 and CA487-C2 are clones from CA487; CA864-A and CA864-B were deduced from the electropherogram of CA864; W837-S43 and W837-S04 are spores from WC837

Heteromorphisms are indicated as follows: K = T/G; M=A/C; R = A/G; W = A/T; Y = C/T

Characteristic nucleotides and positions of sequences of type A or C are in bold type
