# Supplementary material for: Genetic Analyses of the Internal Transcribed Spacer Sequences Suggest Introgression and Duplication in the Medicinal Mushroom Agaricus subrufescens
Source: PLoS One. 2016 May 26;11(5):e0156250. doi: 10.1371/journal.pone.0156250 (PMC4882077; doi:10.1371/journal.pone.0156250)
Supplement: S2 Table — (DOCX) [file pone.0156250.s002.docx]

| Single spore  isolate | Type | Locus | | | | |
| --- | --- | --- | --- | --- | --- | --- |
|  | of ITS | *ITSI* | *ITSII* | *PRS49* | *PRS16* | *PRS88* |
| **Homokaryons** | | | | | | |
| CA487-002 | A | *a* | *-* | *2* | *1* | *2* |
| CA487-006 | A | *a* | *-* | *1* | *1* | *2* |
| CA487-016 | A | *a* | *-* | *1* | *1* | *1* |
| CA487-022 | A | *a* | *-* | *1* | *1* | *1* |
| CA487-023 | A | *a* | *-* | *2* | *2* | *1* |
| CA487-036 | A | *a* | *-* | *2* | *2* | *2* |
| CA487-044 | A | *a* | *-* | *2* | *1* | *1* |
| CA487-049 | A | *a* | *-* | *2* | *2* | *1* |
| CA487-052 | A | *a* | *-* | *2* | *2* | *1* |
| CA487-054 | A | *a* | *-* | *1* | *2* | *1* |
| CA487-057 | A | *a* | *-* | *1* | *1* | *2* |
| CA487-066 | A | *a* | *-* | *1* | *2* | *1* |
| CA487-075 | A | *a* | *-* | *2* | *1* | *2* |
| CA487-078 | A | *a* | *-* | *2* | *2* | *2* |
| CA487-082 | A | *a* | *-* | *1* | *2* | *2* |
| CA487-093 | A | *a* | *-* | *2* | *2* | *2* |
| CA487-095 | A | *a* | *-* | *2* | *2* | *2* |
| CA487-097 | A | *a* | *-* | *2* | *2* | *1* |
| CA487-099 | A | *a* | *-* | *2* | *2* | *1* |
| CA487-001 | B | *b* | *-* | *1* | *1* | *1* |
| CA487-008 | B | *b* | *-* | *2* | *2* | *1* |
| CA487-011 | B | *b* | *-* | *2* | *1* | *1* |
| CA487-024 | B | *b* | *-* | *1* | *1* | *2* |
| CA487-026 | B | *b* | *-* | *1* | *2* | *2* |
| CA487-029 | B | *b* | *-* | *2* | *2* | *2* |
| CA487-030 | B | *b* | *-* | *2* | *1* | *2* |
| CA487-032 | B | *b* | *-* | *1* | *1* | *1* |
| CA487-033 | B | *b* | *-* | *2* | *1* | *2* |
| CA487-035 | B | *b* | *-* | *2* | *1* | *2* |
| CA487-047 | B | *b* | *-* | *1* | *2* | *1* |
| CA487-053 | B | *b* | *-* | *2* | *1* | *2* |
| CA487-060 | B | *b* | *-* | *1* | *1* | *2* |
| CA487-067 | B | *b* | *-* | *1* | *1* | *1* |
| CA487-076 | B | *b* | *-* | *2* | *2* | *2* |
| CA487-077 | B | *b* | *-* | *2* | *2* | *1* |
| CA487-079 | B | *b* | *-* | *2* | *1* | *2* |
| CA487-085 | B | *b* | *-* | *1* | *1* | *2* |
| CA487-087 | B | *b* | *-* | *1* | *1* | *2* |
| CA487-090 | B | *b* | *-* | *1* | *1* | *2* |
| CA487-101 | AC | *a* | *c* | *1* | *1* | *2* |
| CA487-103 | AC | *a* | *c* | *2* | *1* | *1* |
| CA487-010 | AC | *a* | *c* | *2* | *2* | *1* |
| CA487-027 | AC | *a* | *c* | *2* | *2* | *2* |
| CA487-034 | AC | *a* | *c* | *2* | *2* | *1* |
| CA487-037 | AC | *a* | *c* | *1* | *1* | *1* |
| CA487-038 | AC | *a* | *c* | *2* | *1* | *1* |
| CA487-041 | AC | *a* | *c* | *1* | *1* | *2* |
| CA487-042 | AC | *a* | *c* | *2* | *1* | *1* |
| CA487-043 | AC | *a* | *c* | *2* | *1* | *1* |
| CA487-045 | AC | *a* | *c* | *1* | *2* | *2* |
| CA487-046 | AC | *a* | *c* | *2* | *1* | *2* |
| CA487-051 | AC | *a* | *c* | *1* | *1* | *2* |
| CA487-065 | AC | *a* | *c* | *1* | *2* | *1* |
| CA487-069 | AC | *a* | *c* | *2* | *2* | *2* |
| CA487-073 | AC | *a* | *c* | *2* | *1* | *2* |
| CA487-080 | AC | *a* | *c* | *1* | *1* | *1* |
| CA487-081 | AC | *a* | *c* | *1* | *2* | *2* |
| CA487-086 | AC | *a* | *c* | *1* | *2* | *1* |
| CA487-094 | BC | *b* | *c* | *2* | *1* | *2* |
| CA487-096 | BC | *b* | *c* | *1* | *1* | *1* |
| CA487-100 | BC | *b* | *c* | *1* | *2* | *1* |
| CA487-013 | BC | *b* | *c* | *1* | *2* | *2* |
| CA487-014 | BC | *b* | *c* | *2* | *2* | *1* |
| CA487-015 | BC | *b* | *c* | *2* | *1* | *2* |
| CA487-020 | BC | *b* | *c* | *2* | *2* | *1* |
| CA487-031 | BC | *b* | *c* | *1* | *1* | *1* |
| CA487-050 | BC | *b* | *c* | *1* | *1* | *2* |
| CA487-058 | BC | *b* | *c* | *2* | *2* | *1* |
| CA487-064 | BC | *b* | *c* | *2* | *2* | *1* |
| **Putative non-sister nuclei heterokaryons** | | | |  |  |  |
| CA487-070 | A | *a/a* | *-/-* | *1/1* | *2/2* | *1/2* |
| CA487-021 | A | *a/a* | *-/-* | *1/1* | *1/2* | *1/2* |
| CA487-071 | AC | *a/a* | *c/- or c/c* | *2/2* | *2/2* | *1/2* |
| CA487-083 | B | *b/b* | *-/-* | *2/2* | *2/2* | *1/2* |
| CA487-072 | BC | *b/b* | *c/- or c/c* | *1/1* | *1/2* | *1/2* |
| CA487-098 | BC | *b/b* | *c/- or c/c* | *1/2* | *1/1* | *1/2* |
| CA487-088 | BC | *b/b* | *c/- or c/c* | *1/2* | *1/1* | *1/2* |
| CA487-007 | ABC | *a/b* | *c/- or c/c* | *1/1* | *2/2* | *1/2* |
| CA487-019 | ABC | *a/b* | *c/- or c/c* | *1/1* | *2/2* | *1/2* |
| CA487-059 | ABC | *a/b* | *c/- or c/c* | *2/2* | *2/2* | *1/2* |
| CA487-055 | ABC | *a/b* | *c/- or c/c* | *1/2* | *1/1* | *1/2* |
| CA487-074 | ABC | *a/b* | *c/- or c/c* | *1/2* | *2/2* | *1/2* |
| CA487-018 | ABC | *a/b* | *c/- or c/c* | *2/2* | *1/2* | *1/2* |
| CA487-063 | ABC | *a/b* | *c/- or c/c* | *1/1* | *1/2* | *1/2* |
| CA487-084 | ABC | *a/b* | *c/- or c/c* | *2/2* | *1/2* | *1/2* |
| **Putative sister nuclei heterokaryons** | | | | | | |
| CA487-102 | AC | *a/a* | *c/- or c/c* | *1/1* | *1/2* | *1/1* |
| CA487-005 | B | *b/b* | *-/-* | *1/2* | *1/2* | *1/1* |
| CA487-089 | B | *b/b* | *-/-* | *1/2* | *1/2* | *2/2* |
| CA487-092 | BC | *b/b* | *c/- or c/c* | *1/2* | *1/1* | *1/1* |
| CA487-056 | BC | *b/b* | *c/- or c/c* | *1/2* | *1/1* | *2/2* |
| CA487-017 | BC | *b/b* | *c/- or c/c* | *2/2* | *1/2* | *1/1* |
| CA487-091 | AB | *a/b* | *-/-* | *1/2* | *2/2* | *1/1* |
| CA487-039 | ABC | *a/b* | *c/- or c/c* | *1/2* | *1/1* | *1/1* |
| CA487-040 | ABC | *a/b* | *c/- or c/c* | *2/2* | *1/2* | *1/1* |
| CA487-062 | ABC | *a/b* | *c/- or c/c* | *2/2* | *1/2* | *2/2* |
