# Supplementary material for: Genetic Analyses of the Internal Transcribed Spacer Sequences Suggest Introgression and Duplication in the Medicinal Mushroom Agaricus subrufescens
Source: PLoS One. 2016 May 26;11(5):e0156250. doi: 10.1371/journal.pone.0156250 (PMC4882077; doi:10.1371/journal.pone.0156250)
Supplement: S3 Table — (DOCX) [file pone.0156250.s003.docx]

| Locus | Ratio^a^ | Chi-square value |
| --- | --- | --- |
| Mendelian 1:1 ratio tests (1 *df*) | | |
| *PRS49* | 31:38 | 0.72^NS^ |
| *PRS16* | 37:32 | 0.37 ^NS^ |
| *PRS88* | 34:35 | 0.02 ^NS^ |
| *ITSI* | 38:31 | 0.72 ^NS^ |
| *ITSII* | 30:39 | 1.18 ^NS^ |
| Genetic independency tests (1 *df*) | | |
| *PRS49/PRS16* | 41:28 | 2.65 ^NS^ |
| *PRS49/PRS88* | 34:35 | 0.02 ^NS^ |
| *PRS16/PRS88* | 28:41 | 2.43 ^NS^ |
| *ITSI/PRS49* | 32:37 | 0.27 ^NS^ |
| *ITSI/PRS16* | 30:39 | 1.32 ^NS^ |
| *ITSI/PRS88* | 37:32 | 0.38 ^NS^ |
| *ITSII/PRS49* | 33:36 | 0.07 ^NS^ |
| *ITSII/PRS16* | 35:34 | 0.01 ^NS^ |
| *ITSII/PRS88* | 30:39 | 1.15 ^NS^ |
| *ITS1/ITSII* | 30:39 | 1.43 ^NS^ |
